# K_ATP_ channel dependent heart multiome atlas

**DOI:** 10.1038/s41598-022-11323-4

**Published:** 2022-05-05

**Authors:** D. Kent Arrell, Sungjo Park, Satsuki Yamada, Alexey E. Alekseev, Armin Garmany, Ryounghoon Jeon, Ivan Vuckovic, Jelena Zlatkovic Lindor, Andre Terzic

**Affiliations:** 1 Marriott Heart Disease Research Program, Department of Cardiovascular Medicine, Mayo Clinic, Rochester, MN USA; 2 Marriott Family Comprehensive Cardiac Regenerative Medicine, Center for Regenerative Medicine, Mayo Clinic, Rochester, MN USA; 3 Department of Molecular Pharmacology & Experimental Therapeutics, Mayo Clinic, Rochester, MN USA; 4 Department of Biochemistry & Molecular Biology, Mayo Clinic, Rochester, MN USA; 5 Division of Geriatric Medicine & Gerontology, Department of Medicine, Mayo Clinic, Rochester, MN USA; 6 Institute of Theoretical and Experimental Biophysics, Russian Academy of Science, Pushchino, Moscow Region Russia; 7 Mayo Clinic Alix School of Medicine, Regenerative Sciences Track, Mayo Clinic Graduate School of Biomedical Sciences, Mayo Clinic, Rochester, MN USA; 8 Metabolomics Core, Mayo Clinic, Rochester, MN USA; 9 Department of Clinical Genomics, Mayo Clinic, Rochester, MN USA

**Keywords:** Cardiology, Cardiovascular biology, Preclinical research

## Abstract

Plasmalemmal ATP sensitive potassium (K_ATP_) channels are recognized metabolic sensors, yet their cellular reach is less well understood. Here, transgenic Kir6.2 null hearts devoid of the K_ATP_ channel pore underwent multiomics surveillance and systems interrogation versus wildtype counterparts. Despite maintained organ performance, the knockout proteome deviated beyond a discrete loss of constitutive K_ATP_ channel subunits. Multidimensional nano-flow liquid chromatography tandem mass spectrometry resolved 111 differentially expressed proteins and their expanded network neighborhood, dominated by metabolic process engagement. Independent multimodal chemometric gas and liquid chromatography mass spectrometry unveiled differential expression of over one quarter of measured metabolites discriminating the Kir6.2 deficient heart metabolome. Supervised class analogy ranking and unsupervised enrichment analysis prioritized nicotinamide adenine dinucleotide (NAD^+^), affirmed by extensive overrepresentation of NAD^+^ associated circuitry. The remodeled metabolome and proteome revealed functional convergence and an integrated signature of disease susceptibility. Deciphered cardiac patterns were traceable in the corresponding plasma metabolome, with tissue concordant plasma changes offering surrogate metabolite markers of myocardial latent vulnerability. Thus, Kir6.2 deficit precipitates multiome reorganization, mapping a comprehensive atlas of the K_ATP_ channel dependent landscape.

## Introduction

ATP sensitive potassium (K_ATP_) channels operate as high fidelity rheostats in response to metabolic stress^1–5^. Abundant in the cardiomyocyte sarcolemma, where originally discovered^6^, K_ATP_ channels adjust membrane electrical activity to match cellular energetic demand^7,8^. Channel opening under diverse stressor challenges is a recognized cardioprotective event, with channel deficiency associated with poor outcome^9–15^. The K_ATP_ channel dependent molecular landscape, however, remains only partially understood.

Myocardial K_ATP_ channels assemble into a heteromeric complex of the *KCNJ11* encoded Kir6.2 potassium conductive pore and the regulatory ATP binding cassette sulfonylurea receptor 2A (SUR2A) partner^16–18^. Channel metabolic sensing relies on intrinsic ATP mediated gating of Kir6.2, governed by ATP/ADP dependent conformations of tandem SUR2A nucleotide binding domains^19–21^. Under physiological workload, hearts lacking K_ATP_ channels exhibit a switch in metabolic substrate and an augmented oxygen consumption, indicating excessive energy cost compared to hearts containing intact channels^22,23^. Channel linkage to the cellular bioenergetic machinery involves communication with energy shuttles facilitated by near equilibrium enzymatic transfer^24,25^. Messaging with NAD^+^/NADH interconverting enzymes (lactate dehydrogenase), phosphotransferring enzymes (creatine kinase and adenylate kinase), and glycolytic enzymes (glyceraldehyde-3-phosphate dehydrogenase, triose-phosphate isomerase, and pyruvate kinase) have been implicated in K_ATP_ channel contribution to cellular metabolism^26–31^. Comprehensive molecular profiling would enable decoding the full extent of the cardiac K_ATP_ channel interactome.

In this regard, systems biology approaches provide unbiased means of resolving the complex cellular milieu^32,33^. Downstream from genetic and epigenetic inputs, proteomic surveillance captures infrastructure constituents while metabolomic assessment provides a readout of functional activity^34–36^. These complementary approaches facilitate expression analysis and function prioritization based on objective dataset interrogation^37^, and when used in conjunction provide greater insight into complex biological processes than can be achieved from either approach alone. Multiomics data offer extraction of distilled biological signatures, identification of cross-strata common denominators, and merged interpretation. Integrated consideration mitigates misinterpretation due to potential single perspective idiosyncrasy and can alleviate the risk of overlooking pertinent information. These attributes help address the molecular intricacy of the cardiovascular system^38^.

The present study drafts an integrated map of the cardiac plasmalemmal K_ATP_ channel dependent multiome, leveraging a systems strategy applied to a transgenic model lacking the channel pore. Parallel application of proteomics and metabolomics deciphered differential molecular expression segregating Kir6.2 knockout from wildtype hearts. Molecular reorganization induced by K_ATP_ channel loss prioritized a metabo-centric adaptation, handicapped by risk of compromised cardiac resilience. Corroborated in the corresponding plasma metabolome, the resolved multilevel cartography provides an expanded omics guide of K_ATP_ channel reliant cardiac homeostasis.

## Results

### Kir6.2 knockout deviates beyond K_ATP_ channels

**Figure 1:**
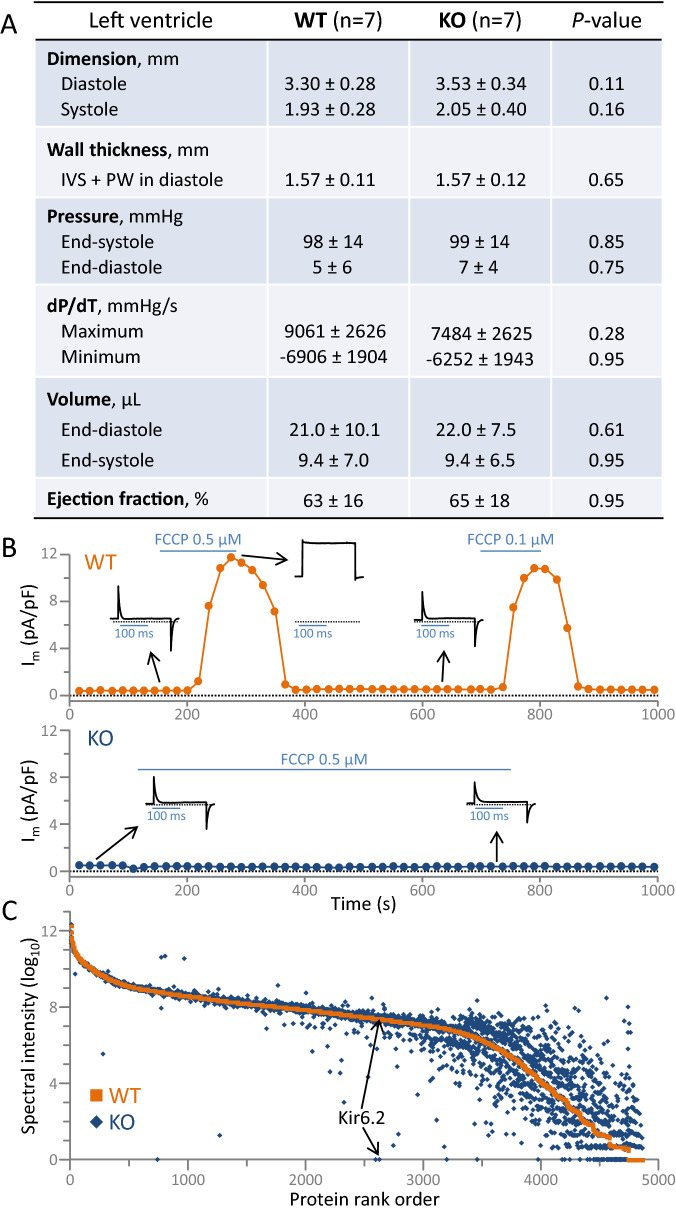
K_ATP_ channel Kir6.2 knockout deviates from wildtype at cardiac proteome level. (**A**) Cardiac ultrasound, left ventricular pressure, and left ventricular pressure–volume conductance showed equivalent chamber size/volume, wall thickness (IVS, inter-ventricular septum; PW, posterior wall), as well as systolic and diastolic function in wildtype (WT) and Kir6.2 knockout (KO). (**B**) In voltage-clamped isolated cardiomyocytes, 2-[2-[4-(trifluoromethoxy) phenyl] hydrazinylidene]-propanedinitrile (FCCP) activated outward current in WT (upper panel; tracing representative of 7 cells) but not in KO (lower panel; tracing representative of 6 cells). Plotted recordings are current values measured at the end of 200 ms long cell membrane depolarization from −40 to −20 mV, normalized to cell capacitance. Horizontal bars represent periods of FCCP application. Insets show whole-cell current recordings prior to and following FCCP application at points denoted by arrows. (**C**) Proteome deviation in KO (blue diamonds; n = 10) is evident as a shift up (increased) or down (decreased) compared to WT (orange squares; n = 10). Individual protein abundance is based on mean ion spectral intensity, plotted for the 4846 detected proteins and rank ordered by WT intensity (average of n = 10), with KO intensity (average of n = 10) at the corresponding x-coordinate. The respective positions of Kir6.2 mean spectral intensity in WT and KO are indicated.

*Kcnj11* ablation produced viable offspring that reached adulthood with no apparent adverse cardiac phenotype at the organ level (Fig. 1A). Adult (3–4 months) Kir6.2 K_ATP_ channel knockout hearts (KO; n = 7) did not differ from age, sex, and environment matched wildtype (WT; n = 7) counterparts on echocardiography and catheterization. Left ventricular end-diastolic/end-systolic dimensions, pressures, volumes, and ejection fraction were all comparable in WT and KO (Fig. 1A). Concordant with K_ATP_ channel ablation, under whole-cell patch clamp, metabolic stress-induced outward current was evident in WT but not in KO cardiomyocytes (Fig. 1B). Mean current density provoked by the oxidative phosphorylation uncoupler 2-[2-[4-(trifluoromethoxy)phenyl] hydrazinylidene]-propanedinitrile was 14.4 ± 1.5 pA/pF in WT (n = 7) versus 0.09 ± 0.08 pA/pF in KO (n = 6) cardiomyocytes (*P* = 0.0002). At the molecular level, high mass accuracy nano-flow liquid chromatography tandem mass spectrometry (LC–MS/MS) of ventricular tissue homogenates (WT, n = 10; KO, n = 10) identified 56,086 peptides assigned to 4846 proteins of which 4205 were quantifiable (Supplementary Table 1). Resolved by label-free relative quantitation (median coefficient of variance: WT = 2.3%, KO = 2.4%), Kir6.2 protein was found abundant in WT but absent in KO (Fig. 1C). In addition to the discrete loss of channel pore expression, extensive KO proteome deviation away from WT was prominent (Fig. 1C). Kir6.2 deletion, while apparently phenotypically silent, causes molecular departure beyond the K_ATP_ channel proper.

### Kir6.2 ablation restructures myocardial proteome

**Figure 2:**
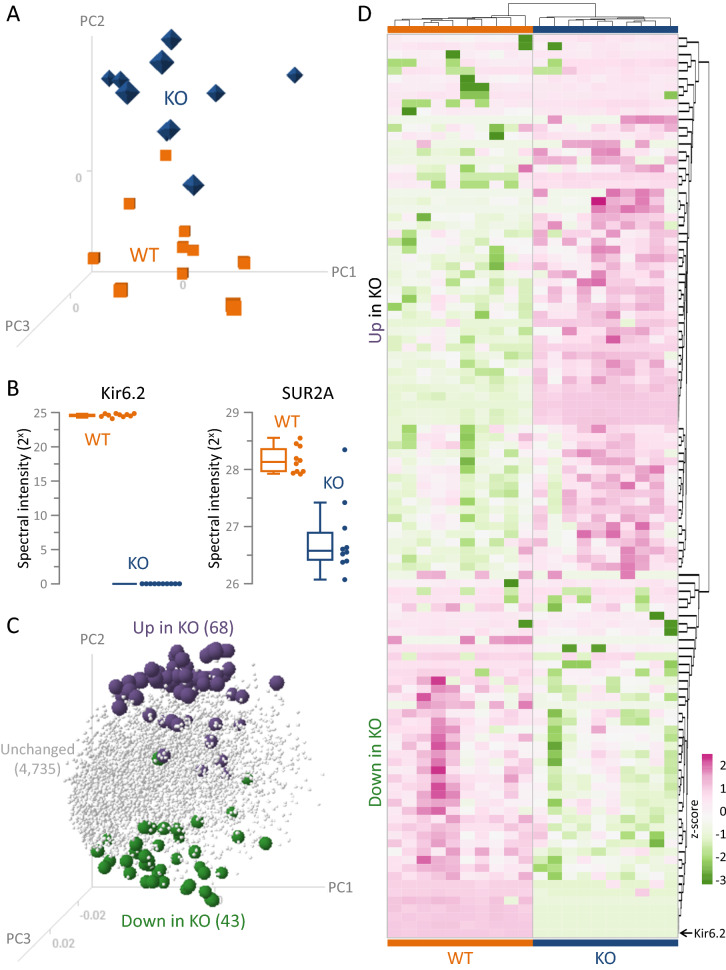
K_ATP_ channel deficient proteome distinguished Kir6.2 knockout hearts. Differential proteomic profiling of wildtype (WT, n = 10) and Kir6.2 knockout (KO, n = 10) ventricular tissue extracts was carried out by data dependent analysis following nano-flow liquid chromatography tandem mass spectrometry. (**A**) KO segregated from WT in singular value decomposition 3-D principal component analysis (PCA), with PC1 representing 22%, PC2 9%, and PC3 7% of the variance yielded from the protein data input. (**B**) Kir6.2 expression was consistent in WT (orange boxplot, orange points) but undetected in KO (blue boxplot, blue points). The K_ATP_ channel partner subunit SUR2A was also reduced (down 1.94-fold, FDR *P* = 0.016) in KO (blue) compared to WT (orange). (**C**) In PCA loading plot of the differentially expressed proteins (with FDR corrected *P* < 0.05), 68 were upregulated (purple spheres, 61.3% of the altered proteome) and 43 downregulated (green spheres, 38.7% of changes) in KO, displaying polar apposition interspersed by unaltered proteins (gray tetrahedrons). (**D**) Clustering by correlation distance and average linkage, a z-score transformed agglomerative heatmap (rose = increased, green = decreased) of differentially expressed proteins distinguished WT from KO (top dendrogram: WT orange, KO blue). The position of Kir6.2 is denoted by an arrow.

**Figure 3:**
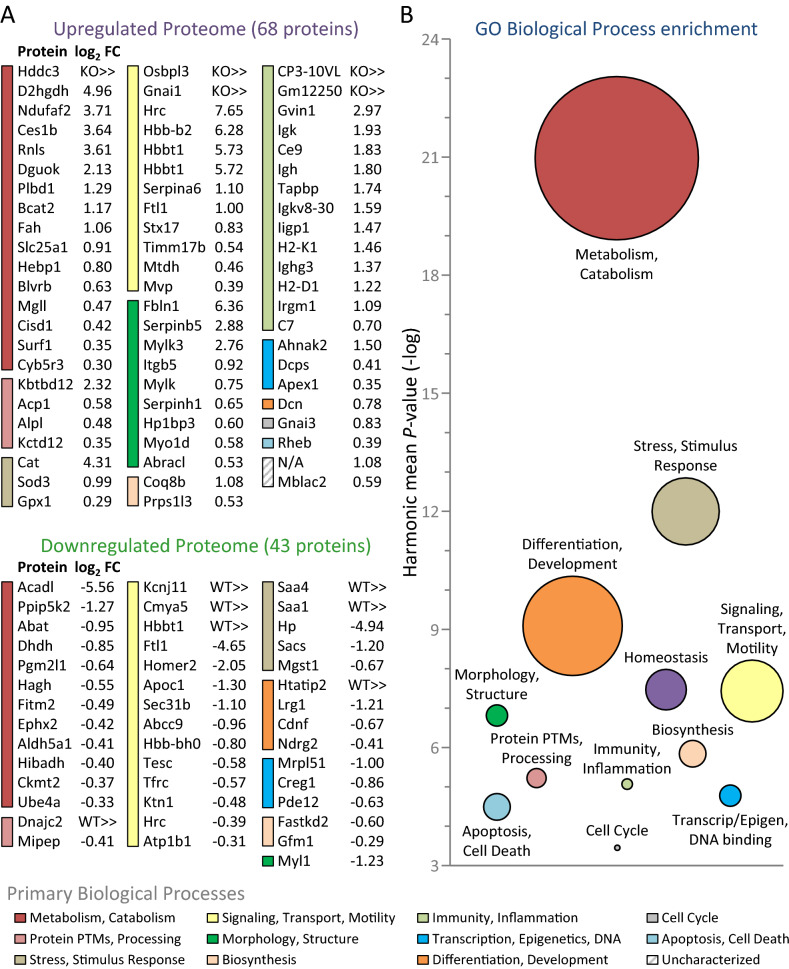
Kir6.2 dependent cardiac proteome spans diverse biological processes and prioritizes metabolic reorganization. (**A**) The 111 proteins significantly altered (FDR corrected *P* < 0.05) in Kir6.2 knockout (KO, n = 10) relative to wildtype (WT, n = 10) heart extracts, including 68 upregulated (upper) and 43 downregulated (lower), are listed by gene symbol with log_2_ fold change (FC) values, and clustered into primary biological process categories from greatest to least extensive change. Proteins denoted ‘KO >  > ’ or ‘WT >  > ’ were detected in 50% or more of the specified cohort and undetected in the other group. Proteome impact was most prominent for ‘Metabolism, Catabolism’ (n = 28 proteins, 16 up and 12 down), followed by: ‘Signaling, Transport, and Motility’ (n = 26, 12 up, 14 down); ‘Immunity, Inflammation’ (n = 14 up); ‘Morphology, Structure’ (n = 10, 9 up, 1 down); ‘Stress, Stimulus Response’ (n = 8, 3 up, 5 down); ‘Protein PTMs, Processing’ (n = 6, 4 up, 2 down); ‘Transcription, Epigenetics, DNA’ (n = 6, 3 up, 3 down); ‘Differentiation, Development’ (n = 5, 1 up, 4 down); ‘Biosynthesis’ (n = 4, 2 up, 2 down); ‘Apoptosis, Cell Death’ (n = 1 up); ‘Cell Cycle’ (n = 1 up); and 2 proteins that remain ‘Uncharacterized’. PTMs = post-translational modifications. (**B**) Bubble plot of the Kir6.2 dependent differential proteome derived network (see Supplementary Figure) prioritized metabolism among enriched biological processes (*P* < 0.001). Enriched biological processes were grouped into distinct clusters (see also Supplementary Table 2). Circle diameters are proportional to the number of enriched biological process annotations per cluster and centered at the harmonic mean *P*-value (−log) for cluster constituents. Calculated as the reciprocal of the arithmetic mean of the reciprocal for all *P*-values in a cluster, the harmonic mean applies Bayesian modeling to account for mutually exclusive *P*-values that are not independent of one another.

Cardiac proteome remodeling imposed by Kir6.2 deletion segregated KO (n = 10) from WT (n = 10) hearts, as visualized by 3-D principal component analysis (PCA, Fig. 2A). Contrasting WT, cardiac plasmalemmal K_ATP_ channel subunits were absent (Kir6.2) or significantly reduced (SUR2A, false discovery rate [FDR] *P* = 0.016) in KO (Fig. 2B). The distinct mitochondrial K_ATP_ channel subunits, Mitok (*Ccdc51*) and Mitosur (*Abcb8*), remained equivalent in WT and KO (see Supplementary Table 1). Of the 4205 quantifiable proteins, 111 were differentially expressed in KO versus WT (limma FDR corrected *P* < 0.05; Fig. 2C). The 68 upregulated and 43 downregulated proteins demarcated a distinct KO molecular substrate delineated by PCA loading plot (Fig. 2C). The resulting agglomerative heatmap distinguished the cohorts based on the differential proteome (Fig. 2D). The Kir6.2 dependent proteome changes spanned 11 primary biological process categories (Fig. 3A). Metabolic or catabolic processes harbored the greatest change, accounting for over 25% of all proteins (28 of 111, with 16 upregulated, 12 downregulated), followed by: signaling, transport, and motility (23%, 12 up, 14 down); immunity or inflammation (13%, 14 up); morphology or structure (9%, 9 up, 1 down); stress or stimulus response (7%, 3 up, 5 down); protein post-translational modification (PTM) or processing (5%, 4 up, 2 down); transcription, epigenetics, or DNA related processes (5%, 3 up, 3 down); differentiation or development (5%, 1 up, 4 down); biosynthesis (4%, 2 up, 2 down); cell cycle (1%, 1 up); apoptosis or cell death (1%, 1 up); with 2 upregulated proteins uncharacterized (Fig. 3A). The spectrum of associated biological processes was validated at the network level, upon integration of the differential proteome within an expanded 239 node neighborhood composed of molecules with known interactions (Supplementary Figure, left). Gene ontology analysis of the network specified 223 associated biological processes enriched at *P* < 0.001 (Supplementary Figure, right, and Supplementary Table 2). Grouping of these processes further highlighted the prioritization of ‘Metabolism, Catabolism’, which harbored the largest proportion of enriched processes (> 27%) and exhibited the greatest extent of significance (−log harmonic mean *P*-value = 20.97) compared to other enriched clusters (Fig. 3B). Thus, metabolism-centric processes dominated the proteome makeover engendered by Kir6.2 ablation.

### Reorganized cardiac metabolome distinguishes Kir6.2 absence

**Figure 4:**
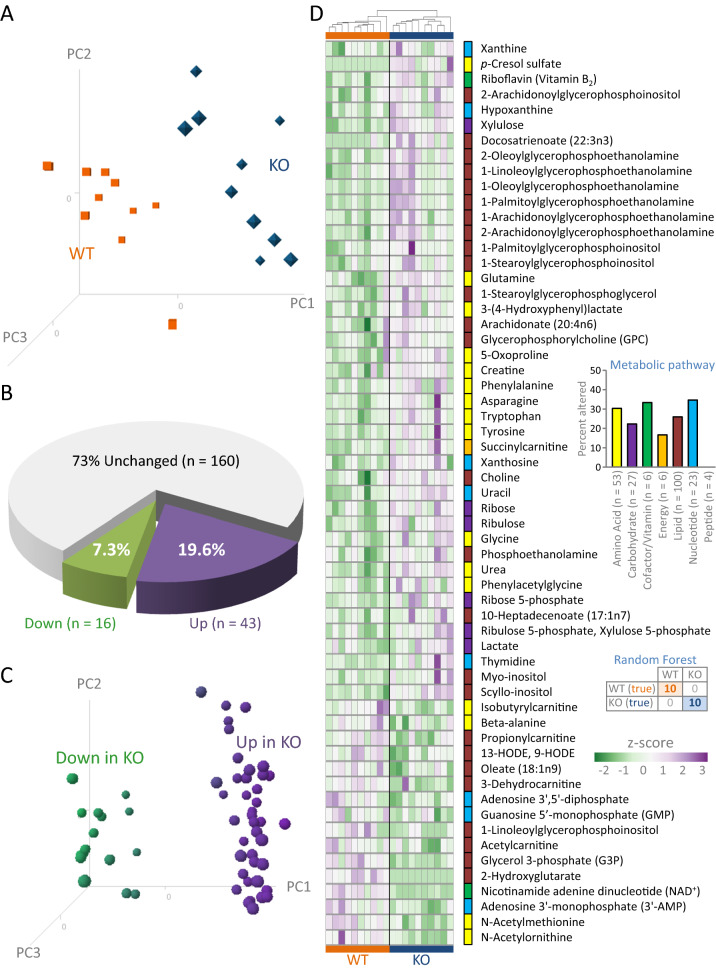
Kir6.2 deletion reforms cardiac metabolome. Differential metabolomic profiling of wildtype (WT, n = 10) and Kir6.2 knockout (KO, n = 10) ventricular tissue extracts was carried out by liquid and gas chromatography mass spectrometry. (**A**) Intra-group clustering and inter-group segregation of WT from KO was evident by singular value decomposition 3-D principal component analysis (PCA), with PC1 representing 44%, PC2 10%, and PC3 8% of the variance yielded from the metabolic data input. (**B**) Of the 219 endogenous metabolites identified, 59 differed significantly between KO and WT (*P* < 0.05), with 43 increased (19.6%) and 16 decreased (7.3%). (**C**) PCA loading plots distinguished differentially up/down expressed metabolites. (**D**) Agglomerative clustering by correlation distance and average linkage of z-score transformed differential metabolites, with differential cohort upregulation (purple) and downregulation (green) in response to Kir6.2 deletion, was distributed across multiple metabolic pathways. Identified metabolites spanned 7 pathway macroclusters (**D upper inset**, with number of detected metabolites in each pathway indicated) with differential expression distributed across 6 of the 7, impacting 17–35% of detected metabolites per pathway macrocluster. Random Forest modeling of the top thirty differential metabolites accurately classified WT from KO (**D lower inset**).

From the WT (n = 10) and KO (n = 10) hearts, distinct metabotypes were independently resolved by high throughput chemometric surveillance using multimodal untargeted mass spectrometry, with prominent cohort segregation evident by 3-D PCA (Fig. 4A). Over one quarter of the measured cardiac metabolome (Supplementary Table 3) was significantly altered by Kir6.2 deletion (59/219 metabolites, *P* < 0.05), with 73% of changing metabolites upregulated and 27% downregulated (Fig. 4B), underscored by differential metabolite loading plots in WT and KO (Fig. 4C). The K_ATP_ channel dependent metabolome, arrayed by unsupervised agglomerative clustering, spanned 6 of the 7 pathway macroclusters encompassing all measured metabolites. Downregulated metabolites contributed to 4 and upregulated metabolites to all 6 pathway macroclusters (Fig. 4D). Kir6.2 deletion precipitated a distinct pattern of change. The percent of metabolites changed in each pathway macrocluster ranged from 17 to 35% (Fig. 4D, upper inset). Specifically, the number of metabolites significantly changed were: 16 (12 up, 4 down) out of 53 in the amino acid cluster; 6 (up) out of 27 in the carbohydrate cluster; 2 (1 up, 1 down) out of 6 in the cofactor/vitamin cluster; 1 (up) out of 6 in the energy cluster; 26 (18 up, 8 down) out of 100 in the lipid cluster; and 8 (5 up, 3 down) out of 23 in the nucleotide cluster. Notably, 100% predictive classification accuracy across cohorts was achieved in Random Forest modeling using the top 30 differential metabolites (Fig. 4D, lower inset). Thus, the resolved chemometric fingerprint mapping the extent and diversity of metabolite changes readily distinguished KO from WT hearts, underscoring the impact of K_ATP_ channel deficiency on the cardiac metabolome.

### Kir6.2 dependent metabolic prioritization

**Figure 5:**
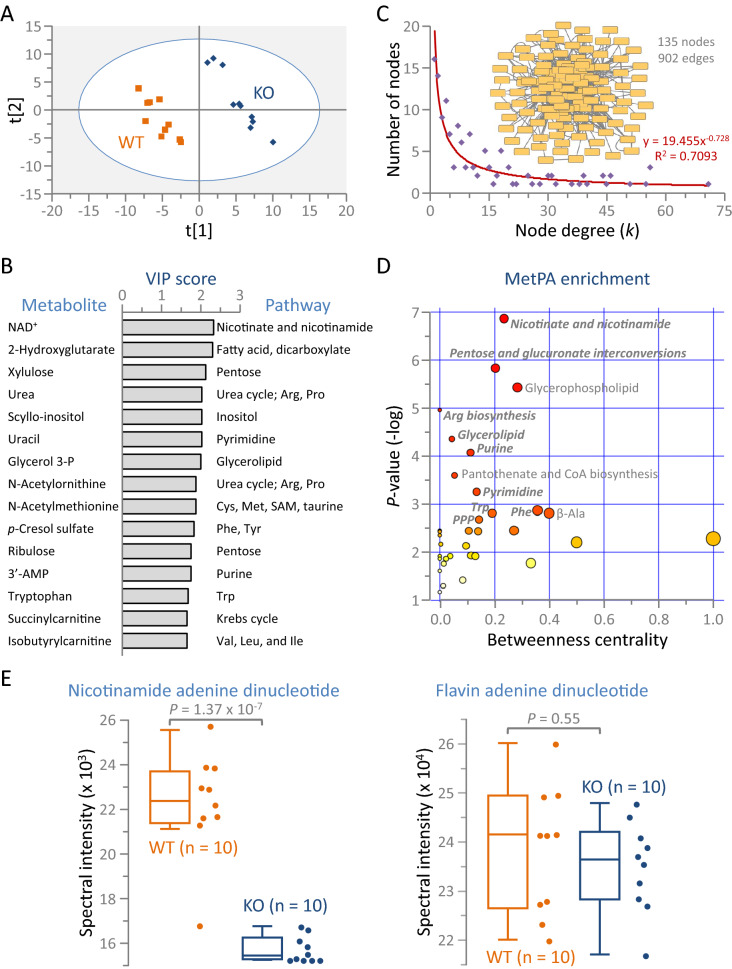
Systems interrogation of the K_ATP_ channel deficient metabolome. (**A**) Supervised classification of the tissue metabolome by partial least squares—discriminant analysis (PLS-DA) segregated wildtype (WT, n = 10) from Kir6.2 knockout (KO, n = 10) hearts, with (**B**) soft independent modelling of class analogy variable importance in projection (VIP) scores ranking nicotinamide adenine dinucleotide (NAD^+^) as the top scoring metabolite for cohort discrimination. (**C**) Pairwise interactions identified by Ingenuity Pathway Analysis for Kir6.2 dependent metabolome changes yielded a 135-node network comprising 902 edges. Network degree distribution (scatter plot) exhibited a scale-free topology, consistent with a biologically structured neighborhood of the K_ATP_ channel deficient metabolome. (**D**) Unsupervised interrogation of the metabolite interactome using MetaboAnalyst Metabolite Pathway Analysis (MetPA) yielded enrichment output synonymous with supervised modeling, independently prioritizing the nicotinate and nicotinamide pathway, while 9 of the 12 most significant pathways (bold italics) overlapped with PLS-DA findings. For each pathway, circle color and size are proportional to *P*-value and betweenness centrality, respectively, with the centrality metric defining differential metabolite contribution to shortest paths within the enriched pathway. (**E**) The significant decrease observed for NAD^+^ in Kir6.2 KO (left) occurred in the absence of change to the related electron acceptor flavin adenine dinucleotide (right). For pathways listed in (**B**) and (**D**): amino acids are indicated by their 3 letter abbreviations; 3-P = 3-phosphate; PPP = pentose phosphate pathway; SAM = S-adenosyl methionine.

**Figure 6:**
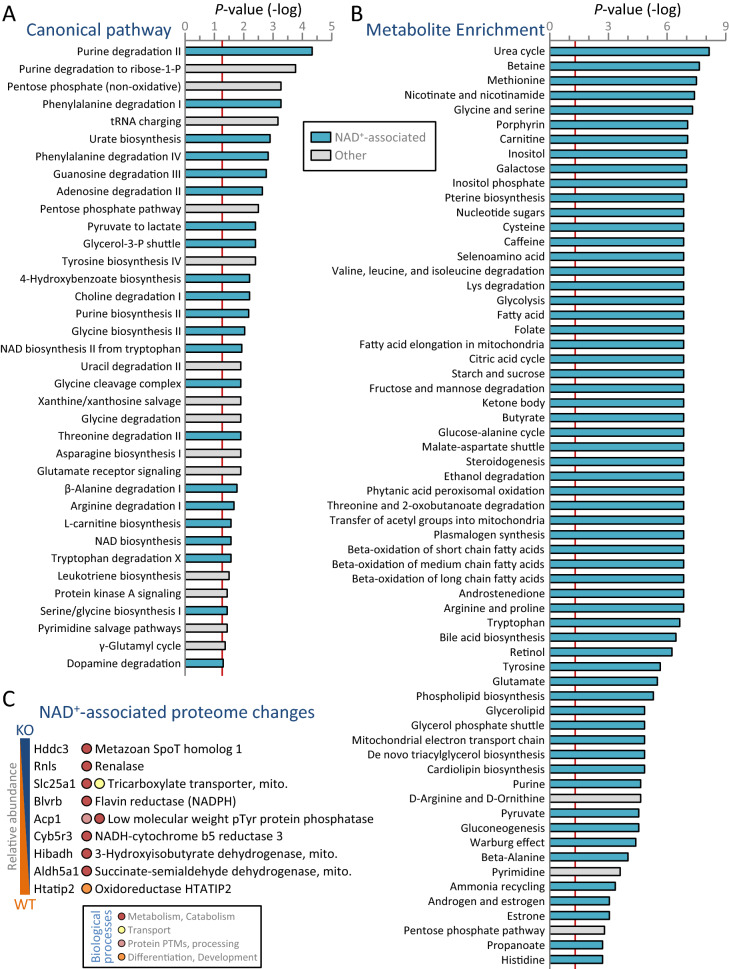
Cross platform validation of NAD^+^-dependent K_ATP_ channel deficiency. (**A**) Enriched pathways arising from the Kir6.2 knockout (KO) versus wildtype (WT) differential metabolome (n = 10 per cohort) were identified in Ingenuity Pathway Analysis canonical pathways (*P* < 0.05) documenting a predominant link to NAD^+^ amongst all 27 differential metabolites associated with overrepresented pathways, with NAD^+^ linking to 22 of 36 pathways and other differentially expressed metabolites linking to 12 or fewer. (**B**) Validating cross-algorithm evidence of NAD^+^ dependence, Metabolite Set Enrichment Analysis specified NAD^+^ linkage to 60 of 63 enriched metabolic pathways (*P* < 0.05), whereas 26 other differential metabolites linked to 9 or fewer pathways each. (**C**) Within the corresponding proteome (n = 10 per cohort), 9 differentially expressed proteins were NAD^+^ associated. Listed by gene symbol, biological processes, and protein name (mito. = mitochondrial), NAD^+^ associated proteins were rank ordered by relative abundance in KO versus WT, with 8 linked to ‘Metabolism, Catabolism’ processes.

Supervised classification of the metabolome by soft independent modeling of class analogy (SIMCA) validated KO and WT intra-group consistency and inter-cohort separation, as evident by partial least squares—discriminant analysis (PLS-DA; Fig. 5A). Systems modeling by SIMCA identified 28 metabolites with variable importance in projection (VIP) scores exceeding 1.5, affirming their prominence in group segregation (Fig. 5B). The top scoring metabolite was nicotinamide adenine dinucleotide (NAD^+^; reduced in KO by ≈ 30% from WT levels). In parallel, nicotinate and nicotinamide metabolism was the top pathway for cohort discrimination. The Kir6.2 dependent differential metabolome was expanded to a 135 node scale-free interactome (Fig. 5C). Unsupervised classification by Metabolite Pathway Analysis (MetPA) of the interactome corroborated the preeminence of NAD^+^ and the nicotinate and nicotinamide pathway (Fig. 5D), with 75% of the most significant MetPA pathways confirmed among the top pathways modeled by VIP scoring (Fig. 5D, bold italicized font). While NAD^+^ levels were significantly reduced in response to Kir6.2 ablation (*P* = 1.37 × 10^−7^; Fig. 5E, left), flavin adenine dinucleotide (the other primary electron acceptor) did not differ between WT and KO cohorts (*P* = 0.55; Fig. 5E, right). Consistent with NAD^+^ prioritization by unsupervised and supervised systems interrogation, NAD^+^ was associated with the greatest number of metabolic and signaling pathways enriched in KO hearts (Fig. 6A,B). Notably, 61% (22/36) of enriched Ingenuity Pathway Analysis (IPA) canonical pathways were NAD^+^ related (Fig. 6A). Less preeminent was glycine linked to 12 enriched pathways, followed by l-glutamine (7 pathways), xanthine (6), l-tyrosine (5), and 4 or fewer IPA enriched pathways for the remaining 22 metabolites. Likewise, 95% (60/63) of enriched Metabolite Set Enrichment Analysis (MSEA) pathways were associated with NAD^+^ (Fig. 6B). In contrast, second-ranked glycine was associated with only 9 of the 63 pathways. Additional metabolites linking to MSEA enriched pathways included l-glutamine (7 pathways), glycerol-3-phosphate (6), and β-alanine (4), with 3 or fewer enriched pathways linking to each of the remaining 21 differential metabolites. Concordant with an NAD^+^-centric KO metabotype, the corresponding Kir6.2 dependent proteome displayed altered expression of 9 proteins associated with NAD^+^ biosynthesis, consumption, or utilization (Fig. 6C). Complementary interrogation thus identified altered metabolites prioritizing key pathways delineating the metabolic identity of the Kir6.2 deficient state.

### Cardiac susceptibility imprinted in the remodeled multiome

**Figure 7:**
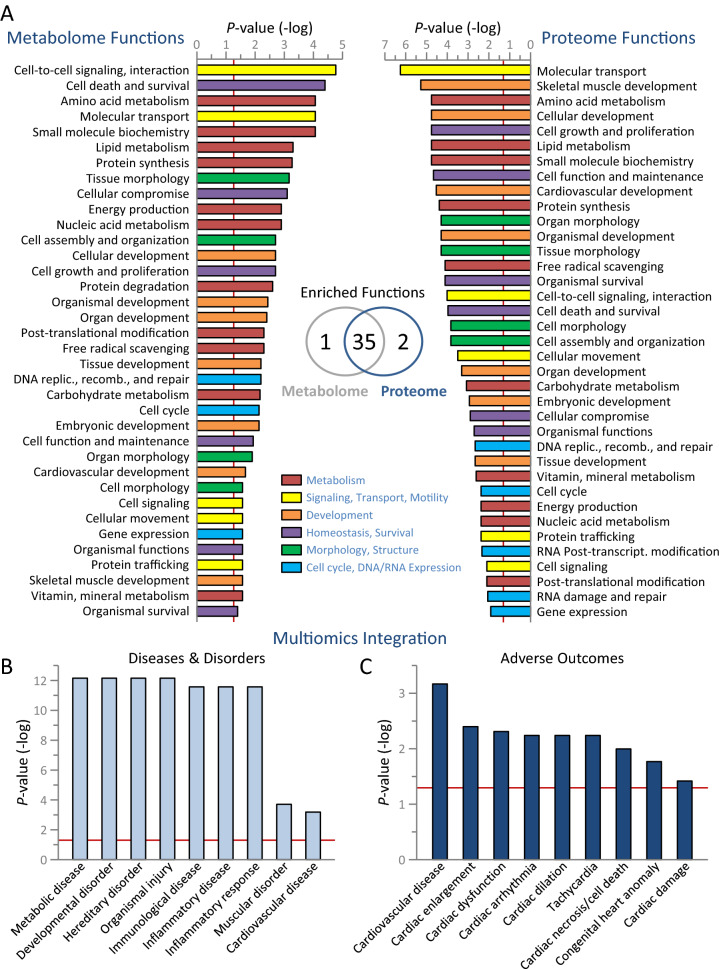
Metabolome and proteome convergence pinpoints acquired disease risk in K_ATP_ channel deficient hearts. Kir6.2 knockout (n = 10) versus wildtype (n = 10) differential metabolome, differential proteome, and merged multiomics integration were interrogated by Ingenuity Pathway Analysis (IPA). (**A**) Differential metabolome enriched cellular functions (left, *P* < 0.05) encompassed metabolism (11 functions), followed in frequency by development (7), homeostasis and survival (6), signaling, transport, and motility (5), morphology and structure (4), and cell cycle, DNA, gene expression functions (3). Differential proteome enriched cellular functions (right, *P* < 0.05) were highly synonymous, with 37 enriched functions matching 97% (35/36) of enriched metabolome functions (center, Venn diagram). Rearranged relative to metabolome ranking, proteome enrichment likewise exhibited a plurality of metabolism functions (10), along with development (7), homeostasis and survival (6), signaling, transport, and motility (5), cell cycle, DNA, gene expression functions (5), and morphology and structure (4). replic. = replication; recomb. = recombination; transcript. = transcriptional. (**B**,**C**) Integrated differential metabolome and proteome data were surveyed in IPA for disease and adverse outcome associations. (**B**) K_ATP_ channel deficiency enriched a collection of diseases and disorders, including metabolic disease, developmental and hereditary disorders, organismal injury, inflammation, and immunological dysfunction, in the context of muscle-related cardiovascular disease. (**C**) The K_ATP_ channel deficient profile predicted susceptibility to cardiac outcomes, with adverse functions ranging across enlargement, dysfunction, arrhythmia, dilation, tachycardia, necrosis/cell death, congenital heart anomaly, and damage.

Integrated multiomics analysis was used to query the influence of the remodeled metabolome and proteome in the setting of Kir6.2 deficiency. Metabolome enrichment profiling in response to Kir6.2 ablation revealed 36 overrepresented functions, prioritizing metabolism (11 functions), followed by development (7), homeostasis and survival (6), signaling, transport, and motility (5), morphology and structure (4), as well as functions (3) involved in cell cycle, DNA, and gene expression (Fig. 7A, left). Of note, 97% of proteome-enriched functions (35/37) matched the metabolome-enriched functions, revealing synonymity across platform readouts (Fig. 7A, Venn diagram). Collective analysis of metabolome and proteome datasets unmasked disease and adverse outcome susceptibility in response to Kir6.2 ablation. Specifically, multiomics interrogation demonstrated an enrichment of metabolic disease, developmental and hereditary disorders, organismal injury, inflammatory and immunological dysfunction, and muscle-related disorder including cardiovascular disease (Fig. 7B). Moreover, an array of cardiac adverse outcomes was overrepresented, with predicted susceptibility to enlargement, dysfunction, arrhythmia, dilation, tachycardia, necrosis/cell death, congenital heart anomaly, and damage (Fig. 7C). Thus, Kir6.2 deficit induces congruent remodeling of the proteome and metabolome, yielding a multiome imprint of cardiac compromise.

### Plasma metabolome distinct in Kir6.2 knockout

**Figure 8:**
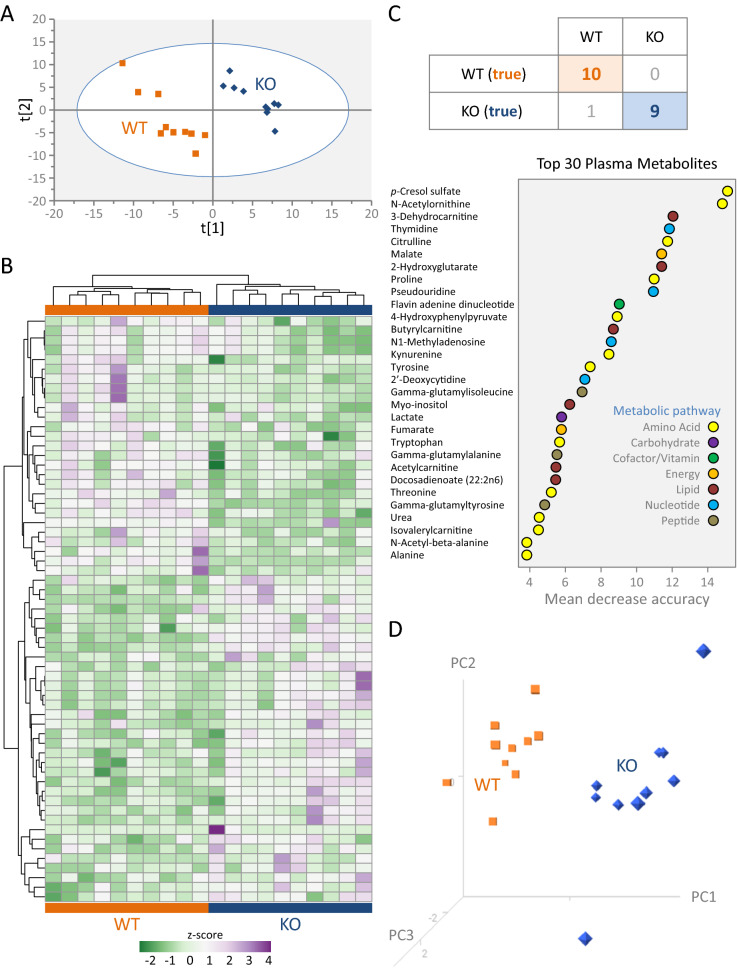
Kir6.2 knockout alters the plasma metabolome. Metabolomic profiling was carried out by liquid and gas chromatography mass spectrometry on plasma from 10 wildtype (WT) and 10 Kir6.2 knockout (KO) animals. (**A**) Supervised classification of the 257 identified plasma metabolites by partial least squares—discriminant analysis documented separation between KO and WT, ranking metabolites based on variable importance in projection scores. (**B**) Nearly one quarter of the resolved plasma metabolome (61/257 metabolites, or 23.7%) was differentially expressed in KO relative to WT, with 34 upregulated (purple) and 27 downregulated (green), separating cohorts by z-score transformed agglomerative clustering by correlation distance and average linkage. (**C**) Random Forest ensembles of decision trees documented 95% accuracy in predictive classification of WT and KO. The 30 differential plasma metabolites exhibiting the largest contribution to classification, rank ordered by mean decrease accuracy scores, spanned metabolic pathways. (**D**) Documenting the discriminatory resolution of differential plasma metabolites, singular value decomposition 3-D principal component analysis segregated KO from WT.

To assess the utility of peripheral plasma in distinguishing Kir6.2 KO, plasma metabolites from corresponding WT (n = 10) and KO (n = 10) mice were isolated and analyzed. Of the 257 measured plasma metabolites (Supplementary Table 4), a quarter (or 61 metabolites) were significantly altered (*P* < 0.05) in response to Kir6.2 ablation. Supervised classification of the plasma metabolome by PLS-DA documented separation of KO from WT (Fig. 8A), with *p*-cresol sulfate and N-acetylornithine the top metabolites in predicting cohort discrimination. Unsupervised agglomerative clustering documented 34 elevated and 27 decreased metabolites, segregating WT and KO cohorts (Fig. 8B). Random Forest modeling achieved 95% predictive classification across cohorts (i.e., correctly allocating 10/10 WT and 9/10 KO; Fig. 8C, upper), and specified *p*-cresol sulfate and N-acetylornithine as top ranked discriminatory metabolites (Fig. 8C, lower). Rank ordered by mean decrease accuracy scores, the top 30 differential plasma metabolites used for classification spanned metabolic pathways (Fig. 8C, lower), with inter-group separation articulated by 3-D PCA (Fig. 8D). Thus, plasma profiling discriminated KO from WT at the metabolome level.

### Distinct Kir6.2 knockout plasma reflects heart metabolome

**Figure 9:**
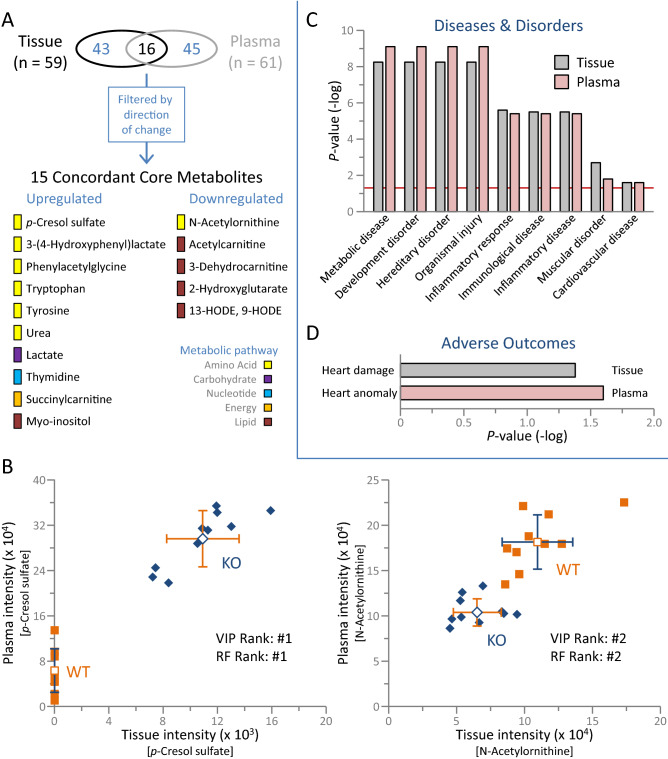
Concordant Kir6.2 knockout plasma and tissue metabolome. (**A**) Overlap between tissue and corresponding plasma of Kir6.2 knockout (KO; n = 10) versus wildtype (WT; n = 10) metabolomes identified 15 shared concordant differential metabolites. Overlapping core metabolites are members of distinct metabolic pathways, primarily associated with amino acid and lipid metabolism. (**B**) WT and KO plasma and tissue spectral intensities were cross-referenced for *p*-cresol sulfate (left) and N-acetylornithine (right), the top ranking metabolites upregulated and downregulated in KO versus WT as modeled by soft independent modeling of class analogy variable importance in projection (VIP) scoring and Random Forest (RF) classification. (**C**) Plasma and tissue Kir6.2 dependent metabolomes yielded matching disease and disorder profiles, with (**D**) equivalent magnitude of predicted cardiovascular adverse outcome.

Functional enrichment analysis of the resolved differential plasma metabolome recapitulated 94% of the 36 functional traits enriched in the corresponding heart metabolome (Supplementary Table 5). Over one quarter of Kir6.2 dependent tissue metabolome changes (16/59) were also detected as differentially expressed in plasma (Fig. 9A, upper). Of these common changes, 94% (15/16) exhibited concordant direction of change in response to Kir6.2 deletion, with 10 upregulated and 5 downregulated metabolites spanning metabolic pathways (Fig. 9A, lower). This shared core included the metabolites prioritized by both SIMCA VIP scoring and Random Forest modeling, namely *p*-cresol sulfate and N-acetylornithine (see also Fig. 8A,C), offering a plasma readout of tissue level change (Fig. 9B). The differential plasma metabolome reproduced the disease and disorder enrichment associations prioritized in the corresponding heart tissue (Fig. 9C). Matching the extent of heart damage susceptibility predicted from the tissue metabolome, the plasma metabolome prognosticated cardiovascular adverse outcome (Fig. 9D). Tissue concordant differential metabolites within the plasma metabolome thus represent potential reporter molecules of latent cardiac susceptibility associated with Kir6.2 deficiency.

## Discussion

The present study demonstrates that hearts deprived of the Kir6.2 K_ATP_ channel pore undergo a proteomic and metabolomic overhaul beyond constitutive channel subunits. The distinct proteome and metabolome conversion underpinned adaptation in hearts lacking functional K_ATP_ channels. Deep phenotyping characterized a metabo-centric metamorphosis across the molecular infrastructure and biochemical output of Kir6.2 devoid hearts, compromised by an imprint of disease susceptibility. The resolved Kir6.2 dependent interactome highlights the centrality of intact K_ATP_ channels in proteome and metabolome maintenance ensuring heart resilience.

**Figure 10:**
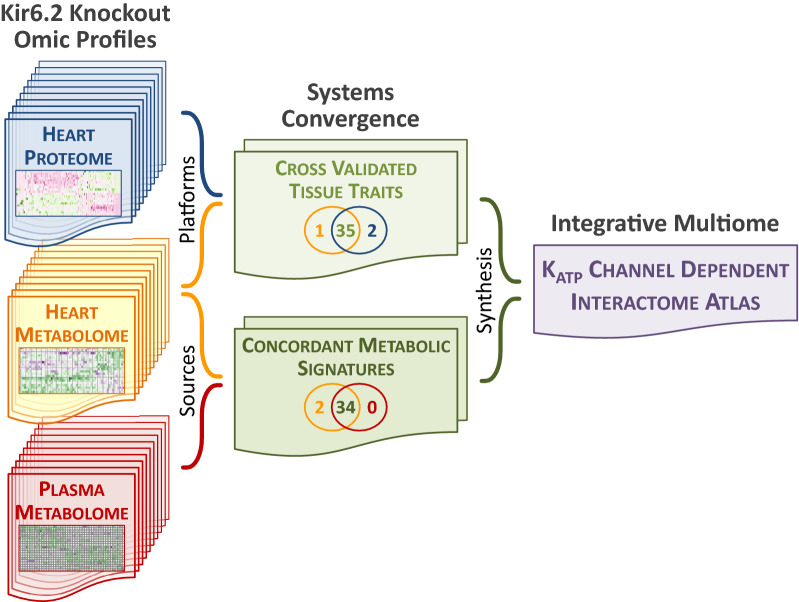
Mapping Kir6.2 dependent multiome atlas. The systems biology approach employed here resolved the Kir6.2 dependent interactome, underscoring the centrality of the intact K_ATP_ channel in heart health. Applied steps included profiling of Kir6.2 knockout (left panels) with the heart proteome (see Figs. 1C, 2 and 3), the heart metabolome (see Figs. 4, 5, 6), and the plasma metabolome (see Fig. 8) sequentially delineated. Systems convergence of multiomics layers (middle panels) details cross-validation of heart proteome and metabolome functional traits (see Fig. 7), while concordant metabolomic signatures are evident between heart and plasma metabolomes (see Fig. 9). Taken together, omic profiles with systems convergence charted a comprehensive atlas of the K_ATP_ channel dependent interactome (right panel).

A systems biology strategy was here employed to acquire and interpret molecular information sampled in vivo across complementary proteomic and metabolomic dimensions^39^ (Fig. 10). Proteomic surveillance of the myocardium identified over 56,000 peptides representing 4846 proteins, enabling untargeted capture of the Kir6.2 dependent expression change spectrum. The high stringency design pinpointed 111 altered proteins across a range of vital cellular processes, demonstrating metabolic primacy of the remodeled K_ATP_ channel deficient heart proteome. Comprehensive protein cataloging extended the findings of more targeted approaches linking metabolism with the cardiac K_ATP_ channel at local partner, associated pathway, or subproteome levels^40–45^. Specificity of observed changes attributed to plasmalemmal K_ATP_ channel integrity was supported by unaltered expression of Mitok and Mitosur, in line with a distinct, non-redundant, channel identity in subcellular compartments^46^.

Underpinnings of metabolic prioritization were further mined by unbiased evaluation of the cardiac K_ATP_ channel dependent metabolome. Multidimensional chemometric profiling revealed that 27% of ventricular metabolites were altered in response to Kir6.2 ablation, spanning metabolic families. The metabolomic changes provoked by Kir6.2 ablation are comparable in magnitude to those characterizing hearts with compromised energy regulators or failing hearts^47,48^.

Notably, Kir6.2 dependent metabolome and proteome enriched functions exhibited remarkable overlap (97% for the metabolome and 95% for the proteome), revealing convergence across platform readouts. Screening multiple omics layers from the same source, in conjunction with data inclusivity free of selection and interpretation bias, supports the validity and utility of considering unique yet interrelated datasets^49,50^. Taken together, the congruent interrogation over multiple molecular strata underscored the impact of K_ATP_ channels as an influential nexus in cardiac metabolism.

Across the breadth of K_ATP_ channel dependent reorganization, systems deconvolution prioritized the multivalent coenzyme NAD^+^ and its associated metabolic pathways. The decrease in NAD^+^ in Kir6.2 deficient hearts was paralleled by change in NAD^+^ associated proteins, including upregulation of NAD^+^ salvage enzymes, namely the metazoan spot homologue 1 (*Hddc3*)^51^ and renalase (*Rnls*)^52^. Maintenance of NAD^+^ is vital to tissue homeostasis^53,54^, with myocardial NAD^+^ pool derangement associated with metabolic remodeling in heart failure and supplementation preserving cardiac performance^55–57^. Notably, NAD^+^ at physiological concentrations regulates K_ATP_ channel activity^58^, and a nicotinamide-rich diet upregulates K_ATP_ channel expression and increases myocardial resilience^59^. In this context, the present findings support a reciprocal relationship of K_ATP_ channels and metabolism, and reveal that the Kir6.2 null heart is typified by NAD^+^ deficit, a prominent feature of cardiomyopathy prone environments^60^.

Indeed, dual metabolome and proteome assessment of the Kir6.2 knockout heart exposed an acquired predisposition to disease susceptibility. This vulnerability signature was herein evident in the young adult at an age apparently free from Kir6.2 dependent extracardiac confounders such as altered insulin secretion, glucose tolerance, and muscle properties^61^. The molecular imprint of heart disease susceptibility was present in advance of overt physiological dysfunction, suggesting that molecular reorganization in response to Kir6.2 deletion is a compensatory adaptation in the young adult animal. Documented independently or collectively across profiling modalities, the current multiomics findings build on initial single omic exploration of Kir6.2 loss^62^. The predictive imprint of disease risk is further reinforced by overt organ failure compromising K_ATP_ channel deficient hearts subjected to stress^63–69^. K_ATP_ channels are implicated in the maintenance of cellular homeostasis, recognized as early responders to metabolic challenge^70^. The mechanism by which Kir6.2 ablation mediates subcellular adaptation needs further study. In principle the observed proteome and metabolome remodelling could be related to the energetically costly KO heart’s propensity for exaggerated Ca^2+^ loading^9,11,12,22^. Calcium overload has been directly implicated in cellular transformation at the protein and metabolite level^71^. Here none of the identified proteins involved in Ca^2+^ handling, regulation, or homeostasis differed in expression between WT and KO (see Supplemental Table 1). This would suggest that omic alterations could be mediated by a proclivity for Ca^2+^ loading on a beat-to-beat basis, rather than a structural change across the Ca^2+^ regulatory proteome.

Corroborating the cardiac disease risk exposed at the tissue level, the resolved K_ATP_ channel dependent plasma metabolome independently reflected myocardial susceptibility. Diverse pathological processes associated with organ failure can be monitored by blood biomarkers, serving as molecular surrogates for early disease diagnosis, stratification, and detection at an asymptomatic state^72^. Among concordant differential metabolites shared between tissue and plasma, *p*-cresol sulfate and N-acetylornithine were consistently prioritized across applied modeling algorithms. Upregulation of *p*-cresol sulfate and downregulation of N-acetylornithine have been associated with cardiovascular disease, namely in (a)symptomatic cardiac dysfunction and incident heart failure^73–76^. These candidate biomarkers offer a clinically applicable and readily accessible source for detecting K_ATP_ channel dependent vulnerability.

Limitations in proteomic and metabolomic analyses may arise from small sample number, restricted data inclusivity, absence of cross-validation, or inadequate application of interrogation resources^77–80^. Here, quality control ensured that the extended cohort size used was adequately powered to capture distinct patterns at high resolution. Moreover, high throughput screening was applied without imposed constraints for inclusive data input, avoiding inadvertent biases. Examining datasets with, and extracting common signatures from, multiple algorithms here provided added confidence in interpretation. Accordingly, supervised and unsupervised approaches were systematically employed following best practices, generating matching output across platforms. Additionally, examination of the heart and plasma in a global deletion model must account for potential confounding effects arising from extracardiac influences. To mitigate this possibility in the present study where Kir6.2 expression in pancreas and skeletal muscle was also impacted, young adult mice (< 4 months of age) were chosen for analysis at an age when insulin secretion, glucose tolerance, and skeletal muscle properties are known to be equivalent between WT animals and those with Kir6.2 deletion^61^.

In conclusion, an atlas of K_ATP_ channel dependent interactome was here constructed using an unbiased systems strategy integrating proteome and metabolome strata. Multiomics surveillance of Kir6.2 null hearts mapped a metabo-centric landscape, exposing latent vulnerability further traceable in the plasma metabolome. The captured multidimensionality of the K_ATP_ channel reliant bioenergetic system offers a broadened perspective on a vital contributor to cardiac homeostasis.

## Methods

### Ethics approval

Protocols were approved by the Mayo Clinic Institutional Animal Care and Use Committee, following National Institutes of Health guidelines. Reporting of animal studies here follows the recommendations in the ARRIVE guidelines^81^. Mice were young adult (up to 4 month-old) male WT (C57BL6) and age-, sex-, environment-matched Kir6.2 null K_ATP_ channel KO counterparts. Of note, up to this age, KO mice maintain insulin secretion, glucose tolerance, and skeletal muscle properties within a normal range^61^.

### In vivo physiology

Group-housed sedentary mice (≤ 5 siblings per cage) received standard chow, with WT and KO exhibiting equivalent glycemic levels^61^. Cardiac structure and function were evaluated under 1–2% isoflurane anesthesia (n = 14). Left ventricular (LV) dimension and wall thickness were measured by echocardiography M-mode parasternal long-axis view (MX400 transducer, Vevo3100 system; MS-400 transducer, Vevo2100; FUJIFILM VisualSonics, Toronto, Canada)^82,83^. Hemodynamics was assessed by LV catheterization (PVR-1045 catheter, MPVS-400; PowerLab 8/30; Miller Instruments, Houston, TX; ADInstruments, Colorado Springs, CO). LV ejection fraction (EF) was calculated as EF% = 100 × (LVEDV − LVESV)/LVEDV, where LVEDV and LVESV are end‐diastolic and end‐systolic volumes^84,85^. First derivatives (dP/dT maximum and minimum) evaluated LV systolic and diastolic pressure^86^. Difference between groups was assessed by Mann‐Whitney *U* test (JMP Pro 14.1.0, SAS Institute Inc., Cary, NC). Data are presented as mean ± standard deviation with *P* < 0.05 significant.

### Cell electrophysiology

Cardiomyocytes were isolated by enzymatic dissociation^87^. Under anesthesia, following thoracotomy, the right ventricle was perfused with 7 mL of HEPES buffer (in mM: 10 4-(2-hydroxyethyl)-1-piperazine ethanesulfonic acid [HEPES], pH 7.8; 130 NaCl; 5 KCl; 0.5 NaH_2_PO_4_; 10 glucose; 10 2,3-butanedione monoxime; 10 taurine) containing 5 mM EDTA. Following aortic clamping, the coronary arteries were perfused through the left ventricle with 10 mL HEPES buffer + 5 mM EDTA, followed by 3 mL HEPES buffer + 1 mM MgCl_2_, and 30–40 mL HEPES collagenase buffer + 1 mM MgCl_2_ (with 0.5 mg/ml collagenase II, 0.5 mg/ml collagenase IV, and 0.05 mg/ml protease XIV). Released cells were filtered through 100 μm nylon mesh and gravity settled, with CaCl_2_ increased to 1.2 mM. Whole cell voltage-clamp was conducted by patch-clamp amplifier (Axopatch 200B, Molecular Devices, San Jose, CA) for cardiomyocytes bathed in (in mM) 136.5 NaCl, 5.4 KCl, 1.0 MgCl_2_, 5.5 glucose, and 10 HEPES–NaOH (pH 7.3) at 31 ± 0.5 °C using an HCC-100A temperature controller (Dagan Corp., Minneapolis, MN). Pipettes (resistance: 4–5 MΩ) contained (in mM) 140 KCl, 1 MgCl_2_, 5 EGTA-KOH, 5 HEPES–KOH, and 5 MgATP (pH 7.3). Stimulation protocol, data acquisition, and cell parameter determination were performed using BioQuest software^88^.

### Multiomics sampling

For molecular profile sampling, excised hearts were rinsed with ice-cold phosphate buffered saline. For proteomics, ventricular apex was placed in cryovials, snap frozen in liquid N_2_, and stored at −80 °C, with remaining ventricle snap frozen and stored at −80 °C for tissue metabolomics. For plasma metabolomics, blood collected in cryovials containing 5 µL of 0.5 M EDTA was centrifuged at 2000×*g* (10 min at 4 °C), with supernatant transferred to fresh cryovials, frozen in liquid N_2_, and stored at −80 °C.

### Proteomics

#### Protein extraction

Ventricular proteins were extracted by 3 rounds of homogenization and centrifugation in 150 µL of 25 mM HEPES, pH 7.4, Mini-Complete™ protease inhibitor (−)EDTA cocktail (Roche Applied Science, Indianapolis, IN), and 1% phosphatase inhibitor cocktails 2 and 3 (Sigma, St. Louis, MO) at 4 °C, followed by 3 rounds of pellet extraction in 150 µL of 7 M urea, 2 M thiourea, and 2% 3-((3-cholamidopropyl) dimethylammonio)-1-propanesulfonic acid^89^. Extracts were quantified by Bio-Rad protein assay (Bio-Rad, Hercules, CA) using bovine γ-globulin standard. Samples (30 µg per extract) were resolved by 10.5–14% gradient Criterion Tris–HCL precast (Bio-Rad) sodium dodecyl sulfate–polyacrylamide gel electrophoresis and stained with Coomassie blue R-250, with gel lanes sectioned for individual mass spectrometry runs.

#### Nano-flow liquid chromatography tandem mass spectrometry

Gel tranches were de-stained, with protein reduced, alkylated, digested with trypsin, and peptides extracted and dried^89^. Peptides were resuspended in 0.2% formic acid, 0.1% trifluoroacetic acid, and 0.002% zwittergent 3–16 (Calbiochem, San Diego, CA), and analyzed by nano-flow LC–MS/MS using a Q-Exactive Hybrid Quadrupole Orbitrap mass spectrometer (Thermo Fisher Scientific, Bremen, Germany) coupled to a Thermo UltiMate 3000 RSLCnano HPLC system. Peptides were loaded onto a 250 nL OPTI-PAK trap (Optimize Technologies, Oregon City, OR) packed with Michrom Magic C8, 5 µm solid phase (Michrom Bioresources, Auburn, CA). Chromatography was performed using 0.2% formic acid in solvents A (98% water, 2% acetonitrile) and B (80% acetonitrile, 10% isopropanol, 10% water), over a 2–45% B gradient for 60 min at 400 nL/min through a 100 µm × 35 cm PicoFrit column (New Objective, Woburn, MA) packed with Agilent Poroshell 120 EC-C18 (Agilent Scientific Instruments, Santa Clara, CA). MS1 survey scans 350–2000 m/z were acquired at 70,000 resolution targeting 3 × 10^6^ ions and 60 ms maximum inject time, followed by data dependent high energy collisional dissociation MS2 on the top 15 ions at 17,500 resolution targeting 2 × 10^5^ ions with 60 ms maximum inject time, using dynamic exclusion of measured ions for 60 s.

#### Mass spectrometry data analysis

Raw files consisting of 10 LC–MS/MS runs per sample were processed in MaxQuant 1.6.7.0^90^, using Andromeda search engine for label-free quantification (LFQ), with applied fastLFQ settings. Spectra were searched against UniProt mouse entries, combining forward and reverse peptides as decoys to estimate FDR, with peptide match and protein assignment FDR set at 0.01. Search parameters included trypsin/P digestion, cysteine carbamido-methylation, and variable modifications of amino-terminal protein acetylation, glutamate to pyro-glutamate, and methionine oxidation. Maximum charge was + 7, with up to 3 dynamic modifications, maximum of 2 missed cleavages, and minimum of 7 amino acids. Mass tolerance was 20 and 10 ppm for first and main searches. LFQ identification was maximized by MaxQuant’s ‘Match Between Runs’ feature, assigning identified spectra from one LC–MS/MS run to corresponding aligned mass and retention time spectra in other runs. Peptides were rolled into protein assignments, requiring ≥ 2 peptides per protein.

#### Differential expression

Relative protein abundance was calculated in R (cran.r-project.org) using Proteus^91^, for limma analysis^92^ of label-free MaxQuant data. Peptide information acquired from MaxQuant evidence files was filtered for contaminants and reverse peptides without imputing missing values. Peptides were rolled into corresponding proteins, data median normalized, and the high-flyer method applied to calculate relative protein abundance. Proteins with FDR corrected *P* < 0.05 were considered differentially expressed.

### Metabolomics

Tissue (> 50 mg) and plasma (> 150 µL) metabolites were processed for untargeted gas chromatography (GC)/MS, and for positive and negative ion mode liquid chromatography (+ LC and − LC)/MS (Metabolon, Research Triangle Park, NC). Protein was removed using organic and aqueous buffers, placed on a TurboVap^®^ (Zymark, Hopkinton, MA), frozen, and small molecules dried under vacuum.

#### Gas chromatography mass spectrometry

For GC/MS of volatile metabolites, samples were re-dried under vacuum prior to derivatization under N_2_ using bistrimethyl-silyl-triflouroacetamide. Samples were analyzed on a Thermo-Finnigan Trace DSQ single-quadrupole MS by electron impact ionization using a 5% phenyl GC column with a 40–300 °C ramp over 16 min.

#### Liquid chromatography mass spectrometry

LC/MS samples were resolved on a Waters ACQUITY UPLC and Thermo-Finnigan LTQ-FT mass spectrometer. For + LC/MS and − LC/MS, extracts were gradient eluted using water and methanol buffers containing 0.1% formic acid or 6.5 mM ammonium bicarbonate, respectively, alternating between MS1 and MS2 injection scans using dynamic exclusion.

#### Identity and expression

Metabolites were identified by matching spectral chromatographic elution properties to Metabolon’s curated library. Expression values were log transformed, and imputation applied using the minimum measured value. Random Forest classification was carried out to model individual cohort allocation, generating decision tree ensembles of the top 30 predictive metabolites. Boxplots of metabolite expression were generated with JMP 14.1.0 (SAS Institute Inc., Cary, NC). Statistical analysis was performed in R, using Welch’s Two-Sample t-test with *P* < 0.05 significant.

### Systems bioinformatics

#### Clustering

Hierarchical agglomerative clustering (with z-score transformed normalization) and PCA visualization were conducted using ClustVis^93^. For 3-D PCA, principal components were plotted in Spotfire 10.0.0 (TIBCO, Palo Alto, CA).

#### Soft independent modeling of class analogy

Metabolome grouping and class membership was predicted by soft independent modeling of class analogy (SIMCA 15.0.2, Sartorius, Bohemia, NY) using PLS-DA. Individual metabolite PLS-DA contributions were rank ordered by VIP scores.

#### Functional enrichment

Differential metabolome interrogation was carried out by MSEA and MetPA within MetaboAnalyst 4.0 (metaboanalyst.ca)^94^. Using Human Metabolome Database (HMDB) identifiers, normalized expression values were analyzed by MSEA, screening Small Molecule Pathway Database (smpdb.ca) libraries. For pathway analysis, the HMDB identifier expression matrix was uploaded in MetPA, surveying the Kyoto Encyclopedia of Genes and Genomes *Mus musculus* metabolic pathway library. In MetPA a global enrichment test was applied, with calculation of the relative betweenness centrality, a network topological parameter of metabolite contribution to shortest paths within the enriched pathway. The entire library served as reference for MSEA and MetPA calculations.

#### Pathway and network analysis

Proteins and metabolites were submitted to IPA (QIAGEN Bioinformatics, Hilden, Germany), prescribing cutoffs of corrected *P* < 0.05 for proteins, *P* < 0.05 for metabolites. IPA output included: enriched canonical pathways; molecular, cellular, and physiological functions; diseases and disorders; cardiac adverse outcomes; and network interactions. Significance was calculated using Fisher’s Exact Test, screening proteins against the gene background and metabolites against the compound library, or both when interpreting merged data. Merged pairwise interactions generated composite networks, exported to Cytoscape 3.8.2^95^. In Cytoscape, NetworkAnalyzer yielded degree distributions to evaluate network topology^96^, with Gene Ontology (GO) Biological Process enrichment assessed in BiNGO (Biological Network Gene Ontology), applying a hypergeometric distribution and Benjamini–Hochberg FDR correction^97^. Enriched processes were clustered and visualized as a bubble plot, with bubble diameters proportional to the number of annotations and vertically centered at the harmonic mean *P*-value^98^.

## Supplementary Information

Supplementary Figure 1.

Supplementary Table 1.

Supplementary Table 2.

Supplementary Table 3.

Supplementary Table 4.

Supplementary Table 5.

## Data Availability

Data supporting findings are available in article/supplementary material.
