# Supplementary material for: KATP channel dependent heart multiome atlas
Source: Sci Rep. 2022 May 5;12:7314. doi: 10.1038/s41598-022-11323-4 (PMC9072320; doi:10.1038/s41598-022-11323-4)
Supplement: Supplementary file 1 — Supplementary Figure 1. [file 41598_2022_11323_MOESM1_ESM.docx]

*Scientific Reports*

**K_ATP_ Channel Dependent Heart Multiome Atlas**

D. Kent Arrell, Sungjo Park, Satsuki Yamada, Alexey E. Alekseev, Armin Garmany, Ryounghoon Jeon, Ivan Vuckovic, Jelena Zlatkovic Lindor, and Andre Terzic

Corresponding Author: Andre Terzic, Mayo Clinic, Rochester, MN, USA (terzic.andre@mayo.edu)

**Supplementary Information**

**Page 2 - Supplementary Figure**

**Page 3 - Abbreviations**

**Supplementary Figure**

**
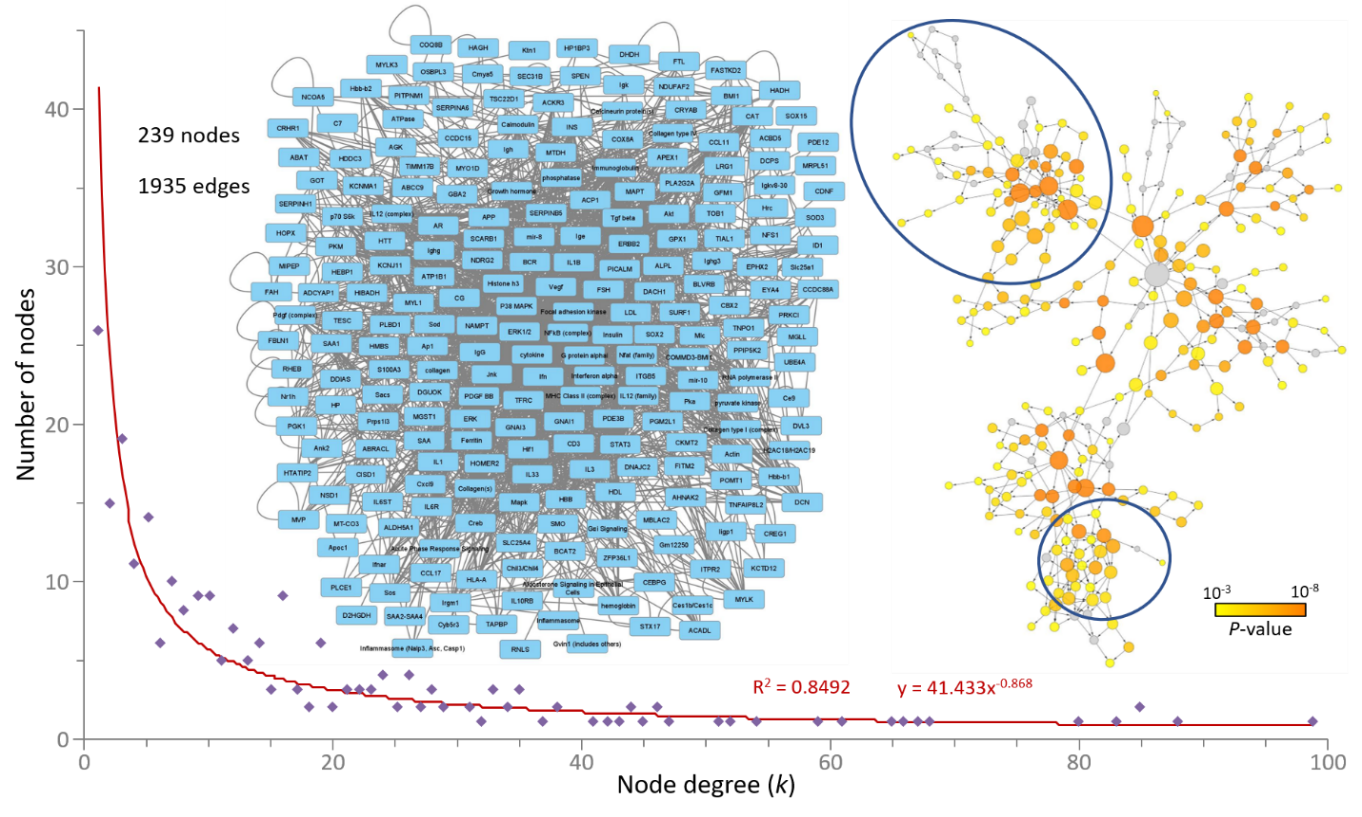
**

**Supplementary Figure.** **Kir6.2 dependent proteome network and enriched biological processes.** Protein identities quantified by limma were uploaded to Ingenuity Pathway Analysis (IPA) for derivation of the composite network integrating the 111 Kir6.2-dependent differential proteins. IPA yielded a 239-node network with 1935 edges, visualized in Cytoscape (left). Network degree distribution (scatter plot) exhibited connectivity consistent with scale-free topology, following a power law distribution (red line, y = 41.433x^-0.868^, R^2^ = 0.85). In scale-free networks, a small proportion of high degree (*k*) nodes serve as hubs, with connectivity much greater than *k*_average_ ([# edges x 2]/# nodes; here *k*_ave_ = 16.19). In turn, BiNGO (**Bi**ological **N**etwork **G**ene **O**ntology) analysis of the proteome network defined a hierarchical network of associated Gene Ontology (GO) biological processes (right). Comprising 270 ontological terms, node size is proportional to number of input proteins linking to the BiNGO term. A total of 223 biological processes were significantly enriched (*P* < 0.001, yellow to orange gradient), with gray nodes not significant. Beyond high level terms overarching the entire GO biological process hierarchy, significant terms represented 1 of 12 specific biological process categories, clustered by bubble plot (Figure 3B). Because visualization of GO terms is not feasible on either the BiNGO network or within the bubble plot, all derived BiNGO network terms are listed and categorized in Supplementary Table 2. The most prominent GO cluster by significance and magnitude, ‘Metabolism and Catabolism’, is represented in two major sectors of the derived BiNGO network (circled regions).

**Abbreviations**

BiNGO Biological Network Gene Ontology

dP/dT first derivative of pressure over time

EDV end‐diastolic volume

EF ejection fraction

ESV end‐systolic volume

FC fold change

FCCP 2-[2-[4-(trifluoromethoxy) phenyl] hydrazinylidene]-propanedinitrile

FDR false discovery rate

GC/MS gas chromatography mass spectrometry

GO Gene Ontology

HEPES 4-(2-hydroxyethyl)-1-piperazine ethanesulfonic acid

HMDB Human Metabolome Database

IPA Ingenuity Pathway Analysis

IVS inter-ventricular septum

K_ATP_ ATP sensitive potassium

Kir6.2 potassium inward rectifier 6.2

KO knockout

LC-MS/MS liquid chromatography tandem mass spectrometry

LFQ label-free quantification

LV left ventricular

MetPA Metabolite Pathway Analysis

MSEA Metabolite Set Enrichment Analysis

NAD^+^ nicotinamide adenine dinucleotide

PCA principal component analysis

PLS-DA partial least squares – discriminant analysis

PTM post-translational modification

PW posterior wall

SIMCA soft independent modeling of class analogy

SUR sulfonylurea receptor

VIP variable importance in projection

WT wildtype
